# Immunohistochemical investigation of prognostic biomarkers in resected colorectal liver metastases: a systematic review and meta-analysis

**DOI:** 10.1186/s12935-018-0715-8

**Published:** 2018-12-27

**Authors:** William Torén, Daniel Ansari, Roland Andersson

**Affiliations:** Department of Surgery, Clinical Sciences Lund, Lund University and Skåne University Hospital, SE-221 85 Lund, Sweden

**Keywords:** Colorectal liver metastasis, Biomarkers, Prognosis, Immunohistochemistry, Tissue microarray

## Abstract

**Background:**

Many studies have investigated the prognostic role of biomarkers in colorectal liver metastases (CRLM). However, no biomarker has been established in routine clinical practice. The aim of this study was to scrutinize the current literature for biomarkers evaluated by immunohistochemistry as prognostic markers in patients with resected CRLM.

**Methods:**

A systematic review was performed according to the PRISMA guidelines. Articles were identified in the PubMed database with selected search terms and by cross-references search. The REMARK quality criteria were applied. Markers were included if they reported the prognostic impact of immunohistochemical markers in a multivariable setting in relation to overall survival (OS). A meta-analysis was conducted when more than one original article provided survival data of a marker.

**Results:**

In total, 26 biomarkers were identified as independent significant markers for OS in resected CRLM. These biomarkers were found to be involved in multiple oncogenic signalling pathways that control cell growth, apoptosis, angiogenesis and evasion of immune detection. Among these biomarker candidates were Ki-67, EGFR, p53, hTERT, CD34, TSP-1, KISS1, Aurora kinase A and CDX2. CD34 and TSP-1 were reported as significantly associated with survival by more than one study and where therefore pooled in a meta-analysis.

**Conclusion:**

A number of independent prognostic biomarkers for resected CRLM were identified. However, most markers were evaluated in a retrospective setting with small patient cohorts, without external validation. Large, prospective, multicentre studies with standardised methods are needed before biomarkers can translated into the clinic.

## Background

Colorectal cancer is the third most common type of malignancy in the Western world and represents a leading cause of death worldwide [1, 2]. Within 3 years of diagnosis, approximately 30% of patients develop metastases located in the liver [3]. For colorectal liver metastases (CRLM) surgical resection is the preferred treatment, providing patients a 5-year survival of up to 60% [4]. However, CRLM is a heterogeneous disease and prediction of individual outcomes after surgery remains a challenge [5]. To improve prognostication in resectable CRLM, much research efforts have been dedicated into prognostic and predictive variables. A scoring system called the clinical risk score has shown to be valuable for estimation of prognosis in several studies [6–8]. Prognostic factors used in this model include resection margin status, extrahepatic disease, node-positive primary colorectal cancer, disease free-interval from primary to metastases, number of hepatic tumours, largest hepatic tumour and carcinoembryonic levels [6]. Over time, several predictive models have been designed to more precisely estimate long-term prognosis using clinical characteristics [9]. Even though these scoring systems have been proven to be useful to some extent, patients with similar risk scores may display varying outcomes. Therefore, a biological approach to stratify patients into risk categories is essential for more accurate prediction of disease outcome.

The molecular transformation of primary colorectal cancer is traditionally reported to involve accumulation of four key mutations, including the oncogenes APC, KRAS, DDC, and the tumour suppressor p53 [10]. Yet, studies have shown that this somatic mutation sequence only occurs in 10% of tumours [11]. Instead, alternative pathways are thought to occur in most cases of primary colorectal cancer [12, 13]. Additionally, further adaptions are required to acquire metastatic capacity and potentiate spread to the liver. The variety of molecular pathways in primary colorectal cancer (CRC) and CRLM may explain the heterogeneity seen both biologically and clinically. Investigation into biomarkers that can enable more precise estimation of prognosis and response to therapy could potentially function as treatment targets and also decrease overtreatment. The benefits of a molecular approach to selection of treatment have been displayed in diseases such as breast cancer [14].

Immunohistochemistry (IHC) is a commonly used staining method where selective antibodies are utilised to quantify and assess distribution of molecular markers in tumour tissue. While other more advanced methods such as quantitative reverse transcriptase-polymerase chain reaction, cDNA microarray and fluorescence in situ hybridization are successively becoming more regularly used in clinical practice, they lack the practical properties of IHC and have not yet become part of routine analysis.

With the increasing interest of biomarkers as prognostic and predictive indicators of outcome, a great number of studies have presented correlations between markers of tumour biology and clinical outcome. However, inter-study differences in methodology, patient characteristics, statistical method and endpoint make the existing data difficult to interpret. The purpose of this study was to summarize the currently available literature on immunohistochemical biomarkers for predicting outcome after liver resection of CRLM.

## Methods

An electronic search of the PubMed database of the National Library of Medicine was executed by the first author to identify all applicable articles. For this systematic review, the PRISMA guidelines were applied [15]. Linked and exploited search terms were ‘colorectal’ ‘hepatic’ ‘liver’ ‘metastasis’ ‘metastases’ ‘prognosis’ ‘survival’ immunohistochem*’. The initial search identified 1073 records. Titles implicating irrelevant subjects were not further studied. Abstracts were screened, and a selection was made based on whether the article could potentially meet the inclusion criteria. Abstracts with relevant content were read in full text to examine eligibility. References identified in the original search where cross-checked for additional eligible articles. The search ended November 2, 2018.

To be eligible for inclusion, the study the biomarkers had to be (1) evaluated in resected CRLM, (2) use IHC, (3) adhere to the REMARK quality criteria [16] and (4) include a minimum of 50 patients. When using tissue microarray (TMA) methodology, the technique had to be described in detail including information about protocols, antibodies, reagents, quantification and interpretation. If more than one articles provided data on the same patient set, only the most recent study was included. No contact was made with authors to collect unpublished data, as this review was limited to records that can be identified through electronic searches in public databases. Studies presented in other languages than English were excluded.

Only studies presenting overall survival (OS) in a multivariable setting, with associated hazard ratio (HR), were included. Additionally, a study had to present 95% confidence interval (CI) and *p* value. If a study did not present a desired parameter, the study was still included if sufficient published data were included to estimate the parameter. If more than one article provided sufficient data of a biomarker, a meta-analysis was performed. The additional calculations and the conduction of meta-analysis were made using Review Manager (RevMan) [Computer program] Version 5.3: The Nordic Cochrane Centre, The Cochrane Collaboration, 2014.

## Results

**Fig. 1:**
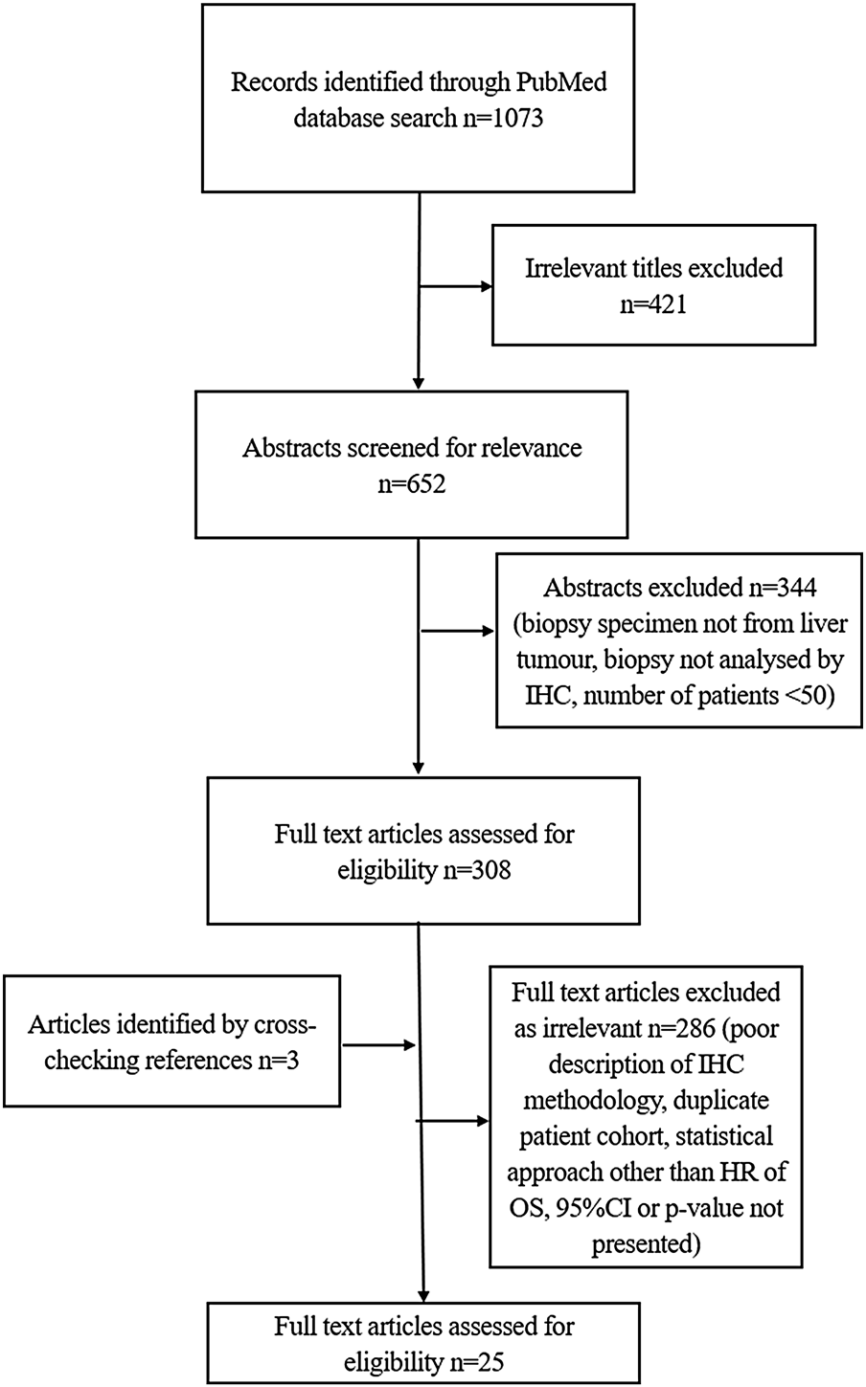
Search strategy

The search strategy is depicted in Fig. 1. A total of 26 biomarkers identified in 25 articles met the inclusion criteria (Table 1). The markers were categorized according to the hallmarks of cancer, as defined by Hanahan and Weinberg [17]: sustaining proliferative signalling, evading growth suppressors, resisting cell death, enabling replicative immortality, inducing angiogenesis, activating invasion and metastasis. Three additional categories were added: deregulated metabolism, controlling the immune system, genome instability (Table 1). Several biomarkers had multiple oncogenic functions, fulfilling criteria for more than one hallmark. In these cases, the markers were categorized in the group according to their most documented mechanism, based on current data of their role in CRLM.

**Table 1:** Independent prognostic biomarkers in resected colorectal liver metastases

Biomarker	References	Year	N	Hazard ratio (95% CI)	Detection rate^a^	p-value
Self-sufficiency in growth signals						
Ki-67	Ivanecz et al. [64]	2014	98	0.82 (0.68–0.98)	27/98 (28%)	0.038
EGFR	Goos et al. [120]	2014	323	1.54 (1.07–2.22)^c^	121/323 (37%)	0.02
RKIP	Kim et al. [77]	2012	68	0.19 (0.09–0.45)^c^	22/68 (32%)	0.014
Insensitivity to anti-growth signals						
p53	Nitti et al. [59]	1998	69	2.53 (1.84–3.22)	44/69 (64%)	0.008
Evading programmed cell death						
TRX-1	Noike et al. [142]	2008	84	0.41 (0.24–0.71)	37/84 (44%)	0.002
FAS/CD95	Onodera et al. [191]	2005	85	3.254 (1.00–10.49)	30/85 (35%)	0.048
Limitless replicative potential						
hTERT	Dômont et al. [40]	2005	201	2.03 (1.46–2.82)	86/201 (43%)	< 0.001
Sustained angiogenesis						
CD34	Miyagawa et al. [131]	2002	71	2.46 (1.13–5.37)	38/71 (54%)	0.023
	Nanashima et al. [132]	2009	139	2.71 (1.15–6.42)	69/139 (50%)	0.023
PTGS2/COX-2	Goos et al. [120]	2014	351	1.59 (1.14–2.26)^c^	85/351 (24%)	0.01
VEGFA	Goos et al. [198]	2016	335	1.50 (1.066–2.111)^c^	101/335 (30%)	0.02
Activating invasion and metastasis						
TSP-1	Sutton et al. [159]	2005	182	1.82 (1.00–3.10)	45/182 (25%)	0.01
	Teraoku et al. [160]	2016	94	0.38 (0.12–0.99)^c^	35/94 (63%)	< 0.05
CAV-1	Neofytou et al. [156]	2017	108	0.40 (0.21–0.78)^c^	61/108 (56%)	0.007
KISS1	Zhu et al. [172]	2015	55	0.20 (0.05–0.91)	19/55 (35%)	0.037
FRZB	Shen et al. [21]	2015	136	2.552 (1.86–3.64)	89/136 (65%)	< 0.001
Deregulated metabolism						
Glucose transporter 1 (GLUT1/SLC2A1)	Goos et al. [198]	2016	350	0.65 (0.51–0.863)^c^	179/350 (51%)	< 0.01
Immune evasion/suppression						
MHC^hi^CD3^hi^	Turcotte et al. [213]	2014	154	0.36 (0.20–0.67)	31/154 (20%)	0.001
CD3+CD8	Wang et al. [212]	2018	249	0.69 (0.59–0.80)	90/249 (36%)	< 0.001
CD45RO	Brunner et al. [211]	2014	201^b^	0.46 (0.28–0.73)^c^	155/201 (77%)	0.001
		2014	201^b^	0.25 (0.10–0.64)^c^	155/201 (77%)	0.004
plgR	Liu et al. [179]	2014	136	2.673 (1.87–3.76)	86/136 (63%)	< 0.001
CD83	Miyagawa et al. [210]	2004	70	0.42 (0.23–0.76)^c^	44/70 (63%)	0.004
Tryptase	Suzuki et al. [209]	2015	135	17.3 (4.80–62)	73/135 (54%)	< 0.01
CD68	Miyagawa et al. [131]	2002	71	2.127 (1.01–4.50)	36/71 (51%)	0.049
Genome instability						
Aurora kinase A	Goos et al. [109]	2013	343	1.66 (1.08–2.54)^c^	115/243 (34%)	0.02
Other markers						
CD133	Yamamoto et al. [91]	2014	103	0.320 (0.13–0.81)	46/103 (45%)	0.016
APOBEC3G	Lan et al. [185]	2014	136	2.582 (1.83–3.63)	91/136 (67%)	< 0.001
CDX2	Shigematsu et al. [217]	2018	396	0.415 (0.26–0.66)	360/396 (91%)	< 0.001

^a^Percentage of samples higher than cutoff

^b^Patient cohort divided into separate analysis

^c^Inverted HR

### Frizzled related protein (FRZB)

FRZB is a negative regulator of Wnt signalling affecting many physiologic functions within the human body [18]. Beta-catenin is one of two pathways that can be activated through Wnt signalling. This pathway is one of the pivotal pathways in stemness and embryonic development. It regulates levels of signalling proteins such as COX-2 and MMP3, which both have documented functions in tumour development [18, 19]. Through this key regulatory function, FRZB has been found as an important oncogene that initiates metastatic properties in human cancers [20]. Approximately two-thirds of CRLM display strong FRZB IHC staining [21], which indicates an upregulation compared to primary CRC [22]. One study analysing the correlation between FRZB and survival in resected CRLM was eligible for this review [21]. In this study, positive IHC staining was found as a significant prognostic factor for poor survival. FRZB was also suggested as a potential candidate target for therapy.

### Human telomerase reverse transcriptase (hTERT)

Telomeres are non-coding repeated DNA sequences localised at the end of each chromosome, protecting it from degradation and chromosomal fusion [23]. The length of the telomere is shortened with each cell division, limiting cellular replicative potential [24–26]. hTERT is one of two functional subunits of telomerase, a reverse transcriptase enzyme with function of maintaining telomere length. hTERT is present in most human cells but is generally repressed resulting in normal chromosomal instability and cellular senescence after a programmed amount of cell cycles [24, 27–30]. Most human cancer attain carcinogenic properties through increased hTERT activity, leading to abnormal replicative potential [31–33]. The significance of telomerase activation has been reported in a wide range of neoplasms, including gastric adenocarcinomas, lung tumours, renal-cell carcinoma and hepatocellular carcinoma [34–37]. These discoveries are coherent with studies reporting a significant correlation between hTERT and decreased OS in primary CRC [38, 39]. In CRLM, the data of hTERT as a prognostic marker is limited, but current data identifies it as an independently significant biomarker for adverse survival [40, 41]. Of these studies, one was found to meet the study inclusion criteria [40].

### p53

The p53 tumour suppressor gene has critical functions in numerous steps of malignant cell transformation. It regulates apoptosis by controlling Bcl2 and Bax [42]. It is involved in DNA repair mechanisms and acts as a cell cycle regulator in the late G1 phase [43]. Also, it regulates TSP-1, which in turn is suggested to have angiogenetic and tumour invasive properties, as describes above [44]. The p53 gene is the most common genetic abnormality found in human cancers [45]. The expression of p53 is reported to be altered in 30–65% of primary CRC [46–56]. There have been reports of increased expression of p53 in CRLM compared to primary CRC [57]. The frequency of p53 alterations in CRLM are described as approximately 65% [58, 59]. Numerous studies have associated altered p53 activity with more advanced stages [46–48, 51, 52, 54, 55] and unfavourable survival in primary CRC [46, 47, 49–56]. The prognostic impact of p53 in CRLM is not as obvious. One study meeting the inclusion criteria found a statistically significant correlation between p53 expression and survival [59]. The association between mutated p53 and decreased survival was confirmed by two studies, however, these studies did not meet the inclusion criteria for this review as other statistical methods were applied [47, 60]. In contrast, several studies investigating the impact of p53 expression on survival found no significant correlation [61–65]. As for the *TP53* gene in CRLM, reports of both significant associations with survival [66–68] and no associations [69, 70] have been presented in studies using other methods than IHC.

### Raf-1 kinase inhibitory protein (RKIP)

RKIP contributes to preserving cells from malignant transformation. It inhibits Raf-1 kinase, an activator of the MAPK signalling pathway which is shown to have an important part in cancer progression [71, 72]. There is also evidence that the MAPK signalling pathway can dysregulate the cell cycle, induce overexpression of VEGF and enable cell mobility through activation of matrix metalloproteinases [73]. In several types of cancer, including primary CRC, reduced expression of RKIP has been associated with advanced cancer stage, metastatic spread and poor survival [74–76]. In primary CRC, RKIP expression has been identified as an independent prognostic risk factor for poor survival [74, 75]. As for resected CRLM, available data demonstrates RKIP as an independent prognostic biomarker for OS [77]. Approximately one-third of CRLM lesions are positive for RKIP [77]. Lastly, RKIP has been suggested to potentiate apoptosis induced by chemotherapy and radiotherapy [78, 79].

### Ki-67

KI-67 is an established marker for cellular proliferation [80]. It is absent in quiescent cells (g0 phase) but is present in cell nuclei during interphase and chromosomes during mitosis [81, 82]. The expression is increased through progression of synthesis phase of the cell cycle [83]. In primary CRC, an association between proliferation and tumour aggressiveness has been displayed [84]. In CRLM, 28–62% of tumours have been estimated as ki-67 overexpressed [64, 85]. Several studies have estimated survival rates in CRLM with proliferation through KI-67 expression. Most data suggest KI-67 overexpression to be of negative impact on survival in patients undergoing resection of CRLM [41, 85–87]. Contrary to this, one study presented an inverse consequence of ki-67 overexpression [64].

### Cd133

Analysis of CD133 is an established method for identifying cancer stem cells, and is currently the most frequently used marker in analyses of human cancers [88]. It is believed that CD133 organizes plasma membrane topology, yet the exact mechanism of action remains unknown [89]. However, more is known of the clinical impact of CD133 status. Expression of CD133 has shown to be of importance in many malignancies, among them primary CRC and CRLM [90, 91]. Lack of CD133 expression was identified as an independent marker for decreased OS after resection of CRLM [91]. One study found an insignificant trend towards CD133-expression and decreased OS after liver resection, although a significant association between CD133 status on disease-free survival was found [92]. Approximately 60% of CRLM lesions have been found to stain positive for CD133 [91]. Furthermore, CD133 is thought to be predictive of chemotherapy response, as expression intensity has been linked to drug resistance [93]. Clinical studies in breast cancer have presented supporting evidence that CD133 has a function in therapy resistance [94], but no studies were found to have presented data of such an investigation in CRLM.

### Aurora kinase A (AURKA)

AURKA regulates the cell cycle by regulating chromosome segregation [95]. The *AURKA* gene is located on a chromosomal region that is often genetically disrupted during primary CRC development and is associated with malign features and poor prognosis [96–102]. When mutated, cell viability, chromosomal stability, growth and invasion is altered [103–107]. Also, evidence suggests that ARUKA mutation initiates, rather than follows these malignant processes [105, 108]. In resected CRLM, ARUKA protein expression has been displayed as an independent prognostic marker for OS [109], which is coherent with previous data that correlates AURKA status to survival in primary CRC [110, 111]. A study showed displayed that AURKA status is concordant in approximately 63% of primary CRC-CRLM pairs [109].

### Epidermal growth factor receptor (EGFR)

EGFR is a receptor known to mediate proliferation and angiogenesis. It acts by acting the MAPK pathway, which is one of the most understood signalling pathways in human cells [112]. Drugs aimed specifically for EGFR have become part of standardised treatment in numerous mutation positive cancers, and is today a included in routine drug regimen in primary CRC [113]. EGFR has an advanced interplay with COX-2, which also functions as a target for therapy. EGFR initiates COX-2 upregulation and COX-2 can potentiate EGFR activation [114–116]. COX-2 inhibitors have found to be a treatment option in anti-EGFR resistant cancers of metastatic CRC [117–119]. In a large patient cohort, EGFR expression has been identified as an independent marker for OS in patients with resected CRLM [120]. The worsening impact of EGFR seems to be of more importance in patients who did not receive chemotherapy. It was therefore suggested that EGFR is one of the targets of commonly used chemotherapy regimen such as 5-FU. Current data suggests that EGFR is an independent prognostic biomarker for survival in resected CRLM [120], however, the optimal use of anti-EGFR agents in treatment of patients with CRLM is not yet concluded. Furthermore, data shows that EGFR status in primary CRC cannot predict EGFR status in CRLM [120–122].

### Cox-2/ptgs2

PTGS-2, also known as COX-2, is one of two cyclooxygenases, converting arachidonic acid to prostaglandin H2. It also promotes PGE2 production, which in turn upregulates cell growth in neoplastic cells [123]. COX-2 inhibitors such as aspirin have been associated with increased survival following resection of primary CRC and decreased incidence of primary CRC overall [119, 124]. As previously described, COX-2 signalling is closely related to EGFR upregulation. In similarity to EGFR, COX-2 overexpression has been found as an independent risk factor for decreased survival in CRLM patients after surgery, especially within patients who did not receive chemotherapy [120]. COX-2 expression is concordant between primary CRC and CRLM [120, 125, 126].

### Cd34

**Fig. 2:**
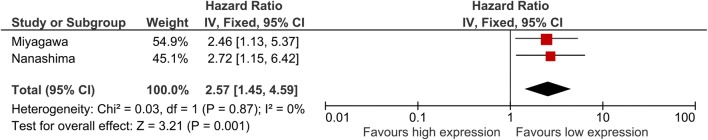
Forest plot of association between CD34 expression and survival after resection of CRLM. A fixed-effect model was used for meta-analysis

CD34 is a frequently used marker for quantification of microvessel density in tumour tissue. Tumour angiogenesis is a fundamental attribute in supporting tumour growth and a hallmark of cancer [17]. The significance of microvessel density in in primary CRC has not been clear. Correlation between a high microvessel density and a poor prognosis has been observed in several studies [127–129]. Contraindicatory, the opposite has also been shown [130]. Two articles have found a correlation between increased staining intensity of CD34 and decreased OS in resected CRLM [131, 132]. Another study found that microvessel density decreases in resected CRLM with standard neoadjuvant chemotherapy [133]. CRLM lesions have been found to have a higher mean microvessel density compared to primary lesions [127]. A meta-analysis was conducted on provided survival data for CD34, which is presented as a forest plot (Fig. 2).

### Thioredoxin-1 (Trx-1)

Thioredoxins are a group of redox proteins that are crucial for human life [134, 135]. Redox activity has been shown as a regulating factor for cellular induction apoptosis and angiogenesis [136]. Current data suggests that Trx-1 levels are increased within cancer cells driven by persistent oxidative stress [137]. Expression of redox proteins is a vital attribute for cancer cells for survival in environments with high oxidative stress [137–139]. Overexpression of Trx-1 has been displayed in numerous forms of human cancer tissue and cancer cell lines, including primary CRC, lymph node metastases from primary CRC and CRLM [140, 141]. Almost 45% of CRLM overexpress Trx-1 [142]. A study presented a significant concordance in staining intensity of Trx-1 between primary CRC and CRLM [142]. High levels of Trx-1 has been associated with decreased survival in primary CRC [141]. In resected CRLM, staining intensity of Trx-1 was found to be an independent prognostic factor for decreased OS by multivariate analysis [142]. Additionally, evidence shows that redox status has a part in cisplatin resistance, a common chemotherapy agent often used in CRLM treatment [143, 144].

### Caveolin-1 (CAV-1)

Awareness of tumour microenviroment as a central component in carcinogenic properties has increased. Interplay between malignant cells and tumour stroma has been recognised as a key component of malignant transformation [145]. One of the most investigated stromal biomarkers is Caveolin-1, a scaffolding protein shown to have a prognostic significance in numerous cancers [146–152], among them primary CRC [152]. Decreased expression of Caveolin-1 has been identified as a main cause of how malignant cells are provided with nutrients [153–155]. One study was found to investigate the role of CAV-1 in survival after resection of CRLM [156]. This study demonstrated decreased stromal CAV1 staining intensity to be a significant biomarker for decreased overall survival by multivariate analysis. A total of 35% of CRLM cells displayed weak CAV1 expression.

### Thrombospondin-1 (TSP-1)

**Fig. 3:**
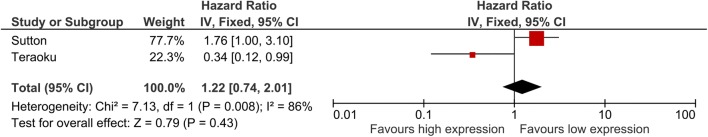
Forest plot of association between TSP-1 expression and survival after resection of CRLM. A fixed-effect model was used for meta-analysis

The exact mechanism of TSP-1 in carcinogenesis remains uncertain. However, many potential functions of have been studied such as thrombocyte aggregation, tissue regeneration, regulation of protease activity and cellular activities such as adhesion, motility, and growth. One study suggested that TSP-1 has both pro- and antiangiogenic properties [157]. Another study showed that cell motility and migration is stimulated by TSP-1 via chemotaxis response [158]. Evidence of TSP-1 as a significant independent marker for OS in CRLM have been presented [159, 160]. Both studies presented significant results, however, one of them found an association between adverse surgical outcome and decreased cytoplasmic TSP-1 [160] and the other one increased stromal TSP-1 to adverse outcome [159]. Interestingly, decreased cytoplasmic expression has been correlated with poor prognosis in cervical [161], lung [162] and breast [163] cancer whilst strong stromal expression has been correlated with poor prognosis in melanoma [164], intraductal papillary mucinous neoplasms [165] and pancreatic carcinoma [166]. Possibly, TSP has different modes of action and a varying significance according to its location. The survival data of TSP-1 was pooled in a meta-analysis (Fig. 3). The different modes of action depending on location of TSP-1 should be noted when interpreting the results presented in the forest plot.

### Kisspeptin 1 (KISS1)

KISS1 is an established tumour suppressor, discovered to be absent in metastatic cells but present in non-metastatic cells [167]. Acquirement of metastatic potential through KISS1 activity has been identified as a central feature in a variety of cancers, including primary CRC [168–171]. Expression of KISS1 has also been correlated with poor survival in human cancers, however, expression patterns differ between different types of malignancies [168–171]. In primary CRC, reduced expression of KISS1 has been suggested as an independent factor for decreased survival and metastatic spread [170]. In CRLM, the impact of low KISS1 expression have been found to be a significant biomarker for decreased OS [172]. The same study found KISS1 expression to be lower in CRLM than in primary lesion. A significant correlation between KISS1 in CRLM and lymphatic spread was also observed.

### Polymeric immunoglobulin receptor (plgR)

plgR is a transporter of immunoglobulins IgA and IgG over epithelial membranes. The expression is strongly promoted by cytokines, thus plgR has a physiologic function as a link between innate and adaptive immunity [173–176]. The clinical impact of plgR in malignant diseases is not fully understood. Increased expression has been detected in many forms of cancer, including primary CRC [177]. Increased circulating levels of plgR has been detected in CRLM [178]. In one study, plgR expression in CRLM tissue was found to be an independent predictor for survival after resection [179]. plgR was also found to indicate high risk of metastatic spread in primary CRC. Almost two-thirds of patients were found to display high staining intensity of plgR in the same study.

### Apobec3 g

APOBEC3G is a regulator of protein synthesis with a central role in anti-viral host defence, especially against HIV [180–183]. Increased expression of APOBEC3G has been documented in CRLM [184]. Two of three CRLM samples has been found to be positive for APOBEC3G [185]. Evaluation of APOBEC3G staining intensity in correlation to survival have identified positive expression as an independent prognostic biomarker in resected CRLM [185]. The same study also suggests that presence of APOBEC3G is a risk factor for metastatic spread in CRLM.

### First apoptotic signal (FAS/CD95)

The FAS receptor, also known as CD95, initiates downstream signalling that results in apoptosis [186]. Stimulation of FAS is one of the two pathways known to induce cellular apoptosis [187]. Resistance to apoptosis has been described as an important part of CRLM progression and other apoptotic markers have been significantly correlated with patient outcome [47, 59, 60, 188]. Also, a correlation between sensitivity to apoptosis and metastatic potential has been suggested [189, 190]. In a study where FAS/CD95 index was analysed in relation to survival after resection of CRLM, it was found to be a strong independent indicator of survival [191]. In the same study, 35% of patients were found to stain positive for FAS.

### Glucose transporter 1 (GLUT1/SLC2A1)

In the malignant transformation of human cells, the metabolism shifts to be more dependent of the anaerobic process glycolysis. This results in an increased demand of glucose. Consequently, an upregulation of membrane glucose transporters has been associated with cancerous properties [192–195]. The expression of GLUT1, also known as SLC2A1, can potentially be regulated by local glucose levels [196]. A meta-analysis found GLUT1 expression to be of importance for survival in solid human tumours, including primary CRC [197]. In CRLM, high expression of GLUT1 was associated with good prognosis [198]. Furthermore, F-FDG PET imaging visualises glucose uptake in tissues via GLUT1 and an increased tracer uptake has been correlated to shorter survival after resection in CRLM [199]. Tumours with a higher metabolic rate can have an increased response to systemic treatment, as cells that are in active phases of the cell cycle are more susceptible to chemotherapy [200–202]. Amplified expression of GLUT-1 could therefore potentially identify patients with higher benefit of treatment.

### Vascular endothelial growth factor A (VEGFA)

VEGFs are a group of proteins that stimulate growth of blood vessels. They are often overexpressed in neoplastic tumours and have been correlated to malign behaviour and decreased prognosis in various types of cancer, including primary CRC [203–205]. Immunotherapy against VEGF has improved survival in patients with primary CRC [206]. In CRLM, neoadjuvant anti-VEGF treatment has been shown to increase rate of radically resected tumours and long-term survival [207]. Increased staining of VEGFA has been proposed as an independent prognostic biomarker for worse survival after resection of CRLM (original). In combination with GLUT1 expression, patients with even worse prognosis could be identified [198]. Furthermore, VEGFA expression seemed to have the most impact on prognosis in patent groups that did not receive systemic chemotherapy (original) which could indicate that conventional chemotherapy affects VEGFA-related processes. Whether VEGFA expression can be used as a predictive marker for anti-VEGF treatment effect is yet to be determined [198, 208].

### Markers of immunologic cells and immunoscores

Analysis of immunologic cells is in cancers is believed to play a significant role in determining tumour aggressiveness. In recent years, the volume of published data of immunologic markers and immunotherapy has remarkably increased. Presence of different cells can indicate properties such as induction of inflammation or evasion of the immune system. Data continuously provide evidence that density of different immunologic cells can function as prognostic markers after resection of CRLM. An accumulation of activated macrophages identified by PG-M1 (antibodies against CD68) was associated with shorter survival after hepatic resection [131]. A high infiltration of masts cells identified with tryptase have been correlated to worse outcome following CRLM resection [209]. A low infiltration of mature dendritic cells has been linked to worse survival [210]. Low presence of CD45RO-positive cells, a marker present in most thymocytes, T-cells and a subset of B-cells, was identified as a risk factor for shorter survival in resected CRLM [211]. Some articles have evaluated immunologic profiling by combining more than one marker to generate so called immunologic scores. A combination of CD3–CD8 (36% detection rate) [212] and high MCH-1CD3 (20% detection rate) [213] independently enable identification of patients with favourable survival. Lastly, it has been suggested that infiltration of immunologic cells may predict response to chemotherapy [214].

### Homeobox transcription factor 2 (CDX2)

CDX2 regulates the maturation of epithelial cells in the gastrointestinal tract. Loss of CDX2 has been shown to negatively impact survival in primary CRC [215]. According to a recent study, the expression of CDX2 is highly concordant between primary CRC and CRLM, independent of whether chemotherapy has been administered prior to primary resection [216]. The prognostic impact of CDX2 expression was investigated in 396 patients with CRLM, where loss of CDX2 expression correlated to decreased survival [217]. CDX2 status has been associated with mismatch repair dysfunction, a hallmark of hereditary CRC (e.g. Lynch syndrome), which is also observed in approximately 10% of sporadic primary CRC [218]. The significance of mismatch repair proteins in CRLM remains to be further investigated.

## Discussion

**Fig. 4:**
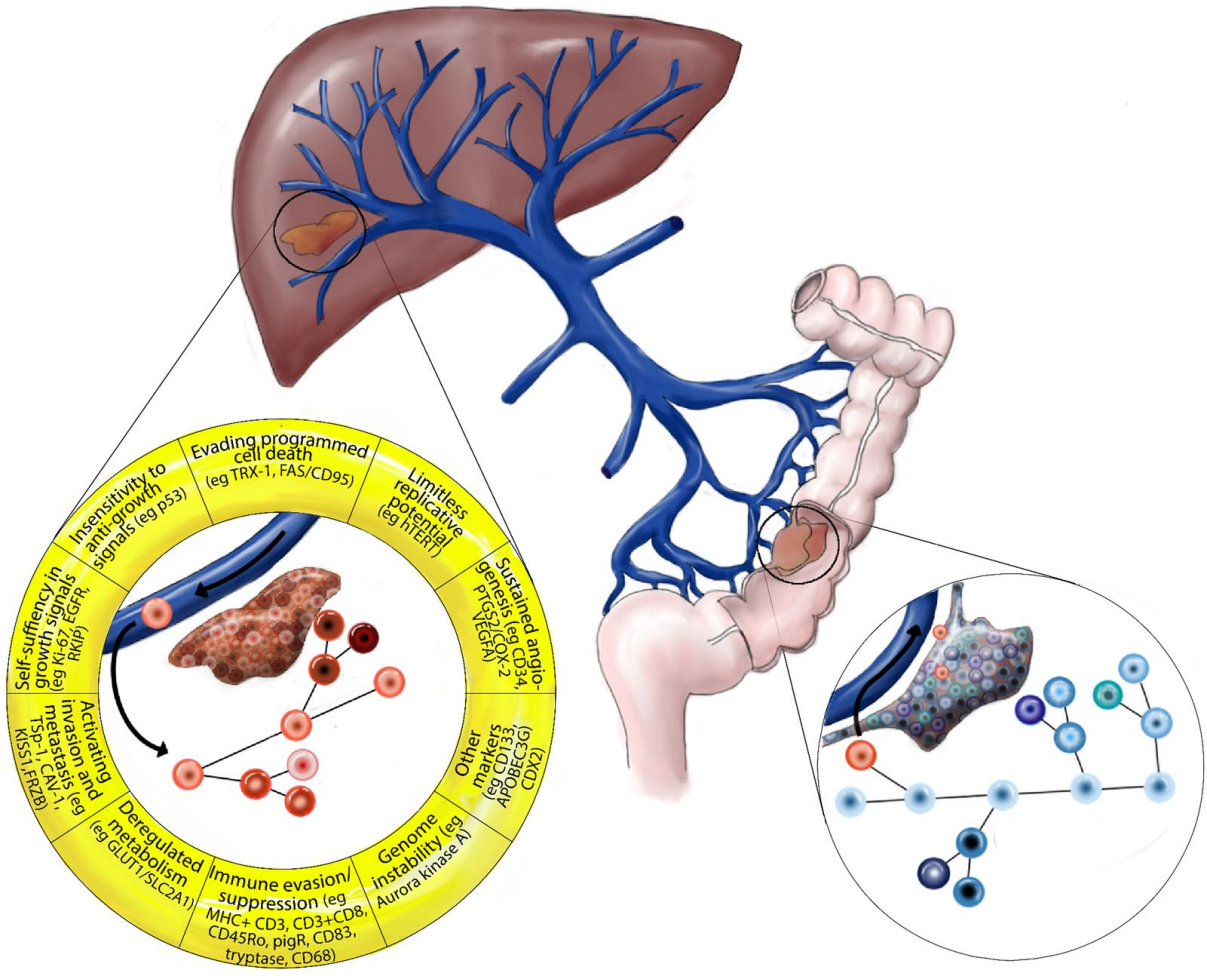
Functional relevance of selected biomarker candidates

This is to our knowledge the first systematic review on immunohistochemical prognostic biomarkers in resected CRLM. We identified 26 independent prognostic biomarker proteins for resected CRLM (Fig. 4). Although IHC/TMA is an effective and established technique for analysing tumour markers, the lack of standardization is a problem. As highlighted by the National Cancer Institute (NCI) and European Organization for Research and Treatment of Cancer (EORTC), issues concerning variations in material (e.g. antibodies), execution (e.g. incubation time) and interpretation (e.g. cut-offs, subjectivity in scoring), are still to be resolved [16]. Additionally, there is no consensus on how survival analyses should be best performed. End points and statistical approach vary between studies and are chosen at the discretion of the authors. Altogether, this makes pooling of studies and conduction of meta-analyses difficult. Subsequently, the likelihood of using biomarkers in clinical practice is impeded by a lack of validation. This highlights the need of prospective multicentre studies with standardised protocols.

Much progress has been made in the molecular pathology of CRLM. A recent study examined the evolutionary relationship between primary CRC and metastases [219]. It was found that CRLM may arise from multiple independent seeding processes, and therefore originate from unique subclones of the primary lesion. It appears that both the primary tumour and the metastases accumulate mutations after metastatic processes have been initiated. This would mean there is heterogeneity not only between patients, but also between the primary tumour and metastasis and even between different metastases within the same patient. Therefore, there could be a diversity in invasive properties and therapy response between malignant cells within a single tumour. The accumulation of mutations in metastatic lesions can lead to discrepancy in protein expression compared to the primary tumour. This is in contrast to previous studies, where it was reported that CRLM maintains protein expression profiles when compared to corresponding primary [220, 221], which seems to be the case only for selected biomarkers. This is important to take into consideration in a future perspective when biomarkers are to be implemented in clinical practice. For example, the decision of whether a patient should receive anti-EGFR therapy for CRLM cannot solely be based on the expression of EGFR in the primary tumour if the expression levels are not corresponding to those in multiple metastases. Furthermore, the complex evolution of primary CRC to CRLM results in many possible mutation cascades, indicating that biological phenotype cannot be judged by only one biomarker. If the prognosis of CRLM is to be better understood and predicted, a panel of biomarkers is required.

Some limitations need to be considered when interpreting this study. Most articles used a retrospective study design, making a reporting and selection bias possible. There was also heterogeneity in the patient cohorts, with variations in factors such as age, gender, ethnicity, comorbidity and tumour characteristics. Methodology of IHC varied. Lastly, most biomarkers lacked validation in external cohorts by independent investigators.

## Conclusions

We identified several independent prognostic biomarkers for resected CRLM. Larger multicentre studies are needed to investigate the real world impact of these biomarker candidates. In the future, these protein markers may potentially be included in biomarker panels to aid in clinical management, such as stratification of patients in risk groups, selection of individual treatments and development of new types of precision drugs.

## Abbreviations

AURKA: Aurora kinase A
CAV-1: Caveolin-1
CDX2: homeobox transcription factor 2
CI: confidence interval
CRC: colorectal cancer
CRLM: colorectal liver metastases
EGFR: epidermal growth factor receptor
GLUT1/SLC2A1: glucose transporter 1
hTERT: human telomerase reverse transcriptase
IHC: immunohistochemistry
KISS1: Kisspeptin 1
OS: overall survival
RKIP: Raf-1 kinase inhibitory protein
TMA: tissue microarray
TRX-1: thioredoxin-1
TSP-1: thrombospondin-1
VEGFA: vascular endothelial growth factor A
